# One-Step Facile Synthesis of Aptamer-Modified Graphene Oxide for Highly Specific Enrichment of Human A-Thrombin in Plasma

**DOI:** 10.3390/s17091986

**Published:** 2017-09-13

**Authors:** Yuan Xu, Siyuan Tan, Qionglin Liang, Mingyu Ding

**Affiliations:** Key Laboratory of Bioorganic Phosphorus Chemistry and Chemical Biology, Ministry of Education, Department of Chemistry, Tsinghua University, Beijing 100084, China; xuyuantsinghua@gmail.com (Y.X.); tsy15@mails.tsinghua.edu.cn (S.T.)

**Keywords:** aptamer, graphene oxide, α-thrombin enrichment, graphene–oxide hybrid material

## Abstract

The enrichment of low-abundance proteins in complex biological samples plays an important role in clinical diagnostics and biomedical research. This work reports a novel one-step method for the synthesis of aptamer-modified graphene oxide (GO/Apt) nanocomposites, without introducing the use of gold, for the rapid and specific separation and enrichment of human α-thrombin from buffer solutions with highly concentrated interferences. The obtained GO/Apt nanocomposites had remarkable aptamer immobilization, up to 44.8 nmol/mg. Furthermore, GO/Apt nanocomposites exhibited significant specific enrichment efficiency for human α-thrombin (>90%), even under the presence of 3000-fold interference proteins, which was better than the performance of other nanomaterials. Finally, the GO/Apt nanocomposites were applied in the specific capturing of human α-thrombin in highly concentrated human plasma solutions with negligible nonspecific binding of other proteins, which demonstrated their prospects in rare protein analysis and biosensing applications.

## 1. Introduction

The enrichment of rare proteins in biological samples is crucial and necessary for applications in therapeutics, disease diagnosis and proteomics [1,2,3]. Although current methods are of high sensitivity in protein determination, it is still challenging to efficiently analyze low-abundance proteins because of their low concentration and the massive interference caused by highly abundant proteins in complex samples. Recently, specific recognition has arisen as a novel approach to achieve such goals. Molecules with affinity tags [4], such as proteins [5], antibodies [6] and aptamers [7], are commonly used for the specific capture and separation of given proteins. Among them, proteins face the problem of the weak binding between proteins, which causes the nonspecific adsorption of other coexisting proteins in complex matrices [8]. Antibodies, although they can specifically bind to target proteins with significant affinity, also have drawbacks, such as their expensive production costs, tedious and complex preparation, easy denaturation and difficult modification [9]. Aptamers, which consist of single-stranded oligonucleotides that can specifically target small molecules [10], proteins [11], viruses [12] and even bacterial and cancer cells [13,14], are a promising alternative [15]. Aside from showing a high affinity equivalent to those of antibodies, aptamers possess several unique features, including small size, facile synthesis, fast amplification and reproduction, good stability, and flexible modification, which make them more attractive for a broad variety of applications than antibodies [16,17,18].

Until now, a wide range of aptamer-modified materials have proven to be effective in rare protein recognition [19]. The materials used for the immobilization of aptamers include silica monoliths [20], sepharose hydrogels [21], magnetic beads [22], activated glass surfaces [23] and gold nanoparticles [24]. These aptamer-modified adsorbents have exhibited an ability for the selective separation and enrichment of trace proteins in various matrices [25]. However, there still remain weaknesses in current methods. Many of these substrates employed to link with aptamers have few functional groups, which means extra processes for their modification are needed; also, their immobilization capacity is insufficient [26]. In addition, time-consuming synthetic steps and rigorous reaction conditions are usually seen in previous studies, leading to a higher complexity and cost of preparation [27]. Furthermore, current substrates have some inherent disadvantages, such as trends of aggregation and hydrophobicity, which make them not only difficult to disperse in aqueous environments, but also suffer undesirable nonspecific adsorption of other proteins [28]. Therefore, it is appealing to develop aptamer-modified adsorbents with facile synthetic procedures, good hydrophilicity and high aptamer immobilization, for protein enrichment in real samples.

Among the various types of substrates, two-dimensional nanomaterials possess many unique characteristics, such as tunable electronic properties, great mechanical strength and flexibility, optical transparency, and a large lateral size with ultrahigh specific surface area, which is extremely attractive for applications in both the electronic and chemical fields [29,30]. Graphene, a two-dimensional material with one-atom thickness, has drawn great attention in materials science and technology [31]. An oxide derivative of graphene, graphene oxide (GO), is considered an ideal adsorbent material in analytical chemistry due to its remarkably large surface area and good water solubility [32]. There exist abundant oxygen-containing groups on both sides of the surface of the GO sheet, such as epoxy, hydroxyl and carboxyl groups, which make GO desirable and easily modified in larger amounts [33]. As a result, GO hybrid materials hold great potential in many biological applications, such as enzymatic research [34], fast proteolysis [35], sensing [36], biological imaging [37] and so on. However, previous studies, which were primarily based on non-covalent modifications like electrostatic interactions and π-π stacking, resulted in inevitably unstable conjugation and low immobilization capacity [38]. Also, the application of modified GO materials as specific protein adsorbents was still in its early stage. Consequently, to develop an easy method for fabricating functionalized GO nanocomposites with high binding capacity and hydrophilicity is very desirable.

In this paper, the one-step facile synthesis of aptamer-modified GO nanocomposites has been achieved, allowing for the efficient separation and enrichment of low-abundance human α-thrombin, which acts as a useful tumor biomarker for the diagnosis of pulmonary metastasis. The obtained nanocomposites not only have good dispersibility in water, but also have a remarkably larger aptamer immobilization capacity than that revealed in previous studies. We have chosen aptamers with three different lengths of spacer molecule to investigate the influence of said spacer molecules on thrombin enrichment efficiency. After pretreatment, the captured protein can be directly analyzed by SDS–PAGE (sodium dodecyl sulfate polyacrylamide gel electrophoresis) without a separate elution step. The ability of the nanocomposites in the specific enrichment of thrombin is also investigated in detail, and the results show that the nanocomposites have higher specific enrichment efficiency of human α-thrombin than the performance of other methods. Moreover, the application of these nanocomposites in real human plasma samples was also achieved.

## 2. Materials and Methods

### 2.1. Chemicals and Materials

Expanded graphite powder (100 mesh) was purchased from Xinghe Graphite Co., Ltd. (Qingdao, China). Chemicals used in the synthesis of GO were purchased from Beijing Chemical Works (Beijing, China). 1-Ethyl-3-(3-dimethylaminopropyl)carbodiimide hydrochloride (EDC) and 2-(N-morpholino)ethanesulfonic acid (MES) were purchased from the J&K (Beijing, China). Bovine serum albumin (BSA) and human α-thrombin were purchased from Sigma-Aldrich. The aptamer targeting human α-thrombin with a five T spacer (5′-TTTTT GGT TGG TGT GGT TGG-3′, named as Apt-15), with the 5′ end modified by the amino group through a C6-carbon spacer arm, was synthesized by Sangong Biotech (Beijing, China). Other chemicals and analytically pure reagents were used as received. All solutions were prepared with deionized water.

### 2.2. Synthesis of Graphene Oxide

GO used in this experiment was synthesized according to a modified Hummers’ method from expanded graphite [39]. Potassium permanganate (36 g) was mixed with ice-cold concentrated H_2_SO_4_ (720 mL) and H_3_PO_4_ (80 mL), followed by the addition of expanded graphite (6 g) slowly, with vigorous agitation to avoid overheating. The reaction lasted 12 h at 50 °C, after which the mixture was transferred into an ice–water mixture of identical volume. The reaction was quenched by adding 30% H_2_O_2_, which turned the slurry into a yellow color, and then rinsing with 10% HCl and water, followed by centrifugation. In the last step, the slurry was purified by dialysis for one week, removing the remaining metal species.

### 2.3. Preparation of GO/Apt Nanocomposites

**Figure 1:**
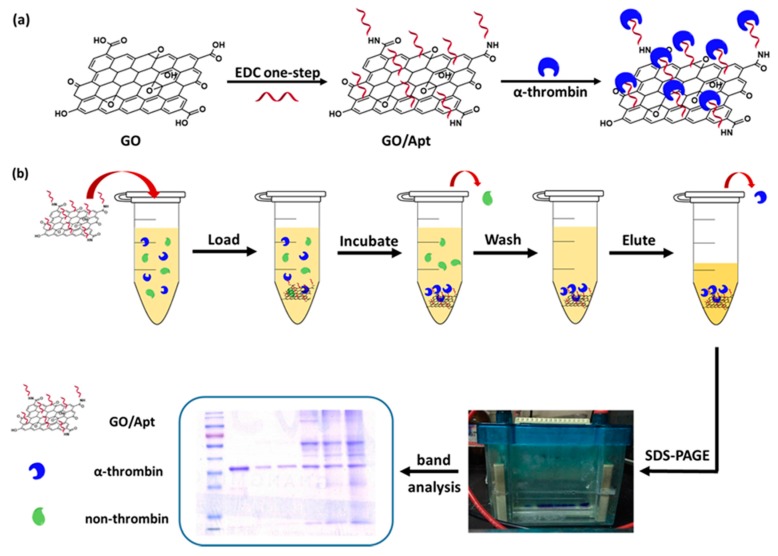
(**a**) Design of the surface immobilization of aptamers on graphene oxide; (**b**) scheme of the α-thrombin-capturing protocol by GO/Apt nanocomposites.

The synthesis of GO/Apt nanocomposites is illustrated in Figure 1a. 300 μg of graphene oxide was dissolved in a 0.1 M MES buffer (pH 4.5) to a final concentration of 1 mg/mL. This solution was suspended by ultrasonication for 30 min.

The aptamer solution (100 μM, dissolved in 0.1 M MES buffer) was added into the GO solution and mixed thoroughly, then the EDC solution (50 mM, dissolved in 0.1 M MES buffer) was slowly added into the mixture through agitation. The reaction was kept at 25 °C and vortexed for 2 h. Then, the solution was centrifuged at 12,000 rpm at 25 °C for 10 min to remove the supernatant that was used for aptamer binding capacity evaluation. The precipitates were washed with deionized water 3 times. Finally, the precipitates were suspended in 300 μL human α-thrombin-binding buffer solution (50 mM Tris-HCl, 140 mM NaCl, 5 mM KCl, 1 mM MgCl_2_, 1 mM CaCl_2_, pH 7.4), and the synthesized nanocomposites were denoted as GO/Apt nanocomposites. The binding capacity of GO/Apt nanocomposites was calculated according to the decrease in the concentration of free aptamers after reaction with the supernatant, using a U-3010 UV spectrophotometer (Hitachi, Japan) at 260 nm.

### 2.4. Human α-Thrombin Enrichment with GO/Apt

Five copies of 20 μg GO/Apt (with 0, 5 and 10 thymidine-modified linkers) were centrifuged at 12,000 rpm for 5 min to remove the supernatant, then mixed with the sample, which consisted of 750 ng human α-thrombin and BSA at five different mass ratios (1:0, 1:10, 1:100, 1:1000 and 1:3000), or which consisted of human plasma at five different concentrations in 100 μL binding buffer solutions, to mimic the complex biological environment. Mixtures were vortexed and incubated for 30 min at room temperature, followed by centrifugation at 12,000 rpm for 5 min, removing the supernatant. The precipitates were washed three times with 100 μL of binding buffer, and then suspended in 10 μL of deionized water. The operation procedure is shown in Figure 1b. Five different volumes of human plasma (with concentrations from 0.03 μL to 300 μL) were diluted into 1 mL of human α-thrombin binding buffer. In the control experiment, 20 μg GO was incubated with the sample above by the same procedure.

### 2.5. Gel Electrophoresis

10 μL of human α-thrombin standards (750 ng), and the GO/Apt solutions with human α-thrombin captured from protein mixture samples, were mixed with 10 μL of gel loading buffer, boiled for 7 min, and loaded onto SDS–PAGE gel. The electrophoresis proceeded at a voltage of 120 V for 1.1 h. After protein separation, the gel was stained with Coomassie blue or silver staining. The obtained gels were scanned with a scanner, and bands in the gels were analyzed by Image J software. The capture efficiency was calculated by comparing the intensity of the experimental bands to the protein standard bands.

### 2.6. Characterization

Transmission electron microscopy (TEM) images were recorded on an H-7650B transmission electron microscope (Hitachi, Japan), zeta potential measurements were carried out on a SZ-100 nanoparticle analyzer (Horiba, Japan) in water, and Fourier-transformed infrared spectra (FT–IR, Perkin Elmer, Waltham, MA, USA) were recorded using a spectrometer in the frequency range of 600–2000 cm^−1^ with a resolution of 4 cm^−1^.

## 3. Results and Discussion

### 3.1. Synthesis and Characterization of GO/Apt Nanocomposites

The synthesis of GO/Apt was accomplished by an amide reaction between the amine groups of the modified aptamers and the carboxyl groups of GO. Compared to the previous method, which used a two-step EDC reaction with the help of sulfo-NHS [40], we found that the one-step EDC modification was much more efficient. During our experiment, it was hard to achieve successful modification through the two-step method. Therefore, we determined that the one-step synthetic strategy is more suitable for the binding of aptamers to the surface of GO.

**Figure 2:**
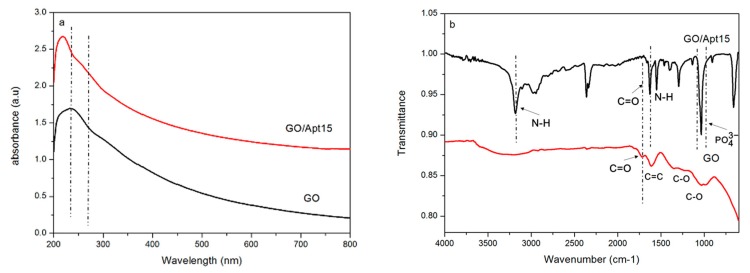
(**a**) UV-Vis spectra of GO and GO/Apt nanocomposites; (**b**) FT–IR (Fourier transform infrared spectroscopy) spectra of GO and GO/Apt nanocomposites.

Zeta potential was first used to demonstrate the successful immobilization of aptamers. As shown in Figure S1, after the one-step reaction done at room temperature, the zeta potential value shifted from −57.28 ± 19.37 mV to −1.67 ± 0.90 mV. Then, both UV-Vis and FT–IR were applied to characterize the successful immobilization of aptamers onto the surface of GO. As shown in Figure 2a, in the UV-Vis spectrum, the GO peaked at around 230 nm, which is consistent with the characteristic π-π* transitions of aromatic C-C bonds. After reacting with the aptamers, the nanocomposites had a new absorption shoulder at 260 nm. This highly specific wavelength was indicative of a nucleotide, owing to the conjugated double bond present in purine and pyrimidine bases. This provided evidence of the presence of aptamers. FT–IR was also applied to examine the forming of covalent bonds between GO and the aptamers, by which successful chemical modification of GO can be confirmed. As shown in Figure 2b, GO had a peak around 1724 cm^−1^, which represents the C=O stretching vibration of the carboxylic group, while both had peaks around 1362 and 1039 cm^−1^, corresponding to the C-O stretching vibrations of the carboxylic group. For GO/Apt, the formation of an amide group was confirmed by the absorption peak around 3181 cm^−1^ for the N-H stretching vibration, and the peak around 1628 cm^−1^ for the C=O stretching vibration. The bending vibration of N-H around 1550 cm^−1^ was also found as evidence of the amide group. In addition, a peak at 1035 cm^−1^ was assigned to the symmetrical stretching vibration of phosphates in nucleotides, supporting the successful immobilization of aptamers onto the surface of GO.

**Figure 3:**
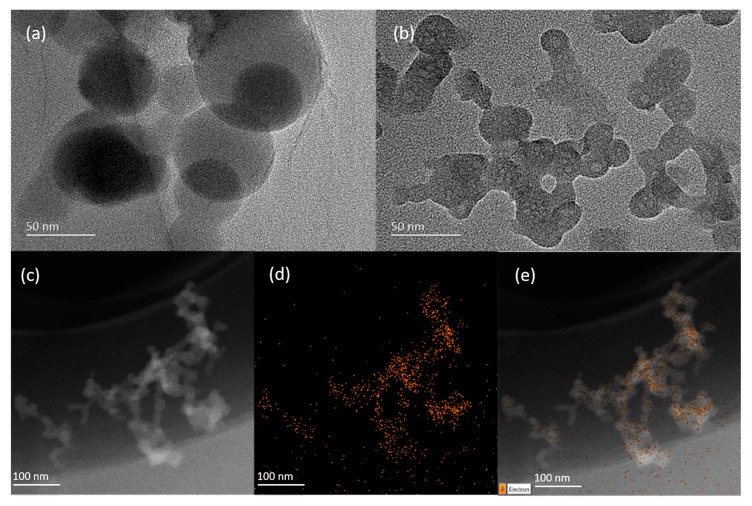
(**a**) TEM (Transmission Electron Microscopy) image of GO; (**b**) TEM image and (**c**–**e**) EDX (energy-dispersive X-ray spectroscopy mapping) results of phosphorus for GO/Apt nanocomposites.

The surface morphology of GO/Apt was characterized by high-resolution TEM. As shown in Figure 3b, the TEM image of GO/Apt revealed that the obtained nanocomposites were irregular in shape at around 100 nm, and they aggregated to form a smaller 2D sheet structure; in contrast, the image of GO—in Figure 3a—shows a relatively large circle-sheet-overlaid structure of about 100 nm in diameter. The results of the TEM images suggested that the GO/Apt nanocomposites formed a new sheet structure with a smaller size and an increased surface area for protein enrichment. The energy-dispersive X-ray spectroscopy (EDX) mapping analysis, shown in Figure 3c–e, revealed the distribution of phosphorus throughout the nanocomposites, which further confirmed the immobilization of aptamers on GO.

The binding capacity of aptamers on the surface of GO was calculated according to the UV absorption value of the supernatant—after the immobilization reaction—at a wavelength of 260 nm, which indicated the amount of aptamers remaining in solution that did not react with GO. The difference from the initial aptamer sample would be immobilized aptamers. The binding capacity calculated was as high as 44.8 nmol/mg (RSD = 1.93%, *N* = 5), which was significantly beyond that of π-π adsorption on GO (50 pmol/mg) [41] and Fe_3_O_4_@C@Au magnetic microspheres (0.15 nmol/mg) [42], and even higher than Au/PEI/GO (gold functionalized graphene oxide nanocomposites) nanocomposites (36.1 nmol/mg) [43]. This high efficiency should be attributed to two aspects: firstly, there were plenty of carboxylic acid groups on the surface of GO for the immobilization of aptamers, and the ultra-high surface area of GO also made it possible to react with amino-modified aptamers thoroughly; and secondly, the highly efficient one-step reaction was also considered to promote the binding capacity.

### 3.2. Enrichment and Recognition of Proteins by GO/Apt Nanocomposites

Human α-thrombin was chosen as a model analyte to evaluate the adsorbing performance of the GO/Apt nanocomposites, and these experiments were conducted by the SDS–PAGE technique because the introduced SDS solution could efficiently disrupt the interaction between the protein and aptamers. This would lead to a simple release of protein captured by the GO/Apt nanocomposites for gel electrophoresis, without an extra elution step.

**Figure 4:**
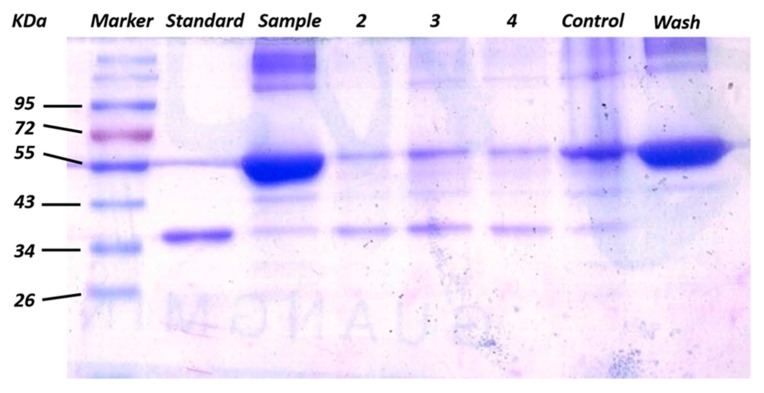
Gel electrophoresis results of captured α-thrombin via GO/Apt nanocomposites from 750 ng of α-thrombin spiked in the buffer solution of BSA with a mass ratio of 1:100 with 0, 5 and 10 thymidine linkers (lanes 2–4). Standard lane represents 1 μg α-thrombin. Control lane represents 20 μg GO incubated with 750 ng α-thrombin spiked in the buffer solution of BSA in the same mass ratio. Sample lane represents 10 μL of mixture to be enriched. Wash lane represents 10 μL of washed solution after GO/Apt enrichment.

The adsorbing performance of GO/Apt nanocomposites toward the target protein could be influenced by linkers between aptamers and the GO surface. Steric hindrance between immobilized aptamers decreased as the length of the linkers increased, making it easier to fold into tertiary structures and access the target protein. Balamurugan et al. demonstrated two factors that mainly affect the efficiency of capture: surface capacity, and the distance of aptamers toward the immobilization surface [44]. In our study, aptamers with three different lengths of thymidine linker (T_0_, T_5_ and T_10_) were used to investigate and optimize the effect on capturing efficiency. 20 μg of GO/Apt nanocomposites, with the zero-, five- or ten-length thymidine linker-modified thrombin aptamers, were incubated with 750 ng human α-thrombin-spiked buffer in the presence of BSA (66 KDa) (which was 100 times more concentrated than α-thrombin (37 KDa)), and the capturing efficiency comparison experiments were then conducted by an SDS–PAGE technique. As shown in Figure 4, as the linker length increased from T_0_ (lane 2) to T_5_ (lane 3), the capture efficiency increased from 58.4% to 85.1%. However, the capture efficiency decreased back to 58.4% when the linker increased from T_5_ to T_10_ (lane 4). Therefore, the aptamers with the T_5_ thymidine linker presented the maximum efficiency of thrombin capturing. These results concluded that at T_5_, the distance effect was the most suitable for maximizing the efficiency of thrombin capture. GO/Apt nanocomposites with the T_5_ linker were used in the following experiments as well. The control lane represents the result of protein captured by GO, which has a nonspecific protein capturing ability. The protein captured by GO was mainly BSA, which was much more intensified than GO/Apt. Meanwhile, the band intensities for captured α-thrombin were so low that the capture of α-thrombin by GO could be considered negligible. Compared with lane 2, which represented the sample of mixed protein in which BSA is 100 times more concentrated than α-thrombin, it could be demonstrated that the human α-thrombin was successfully captured and enriched by GO/Apt nanocomposites.

**Figure 5:**
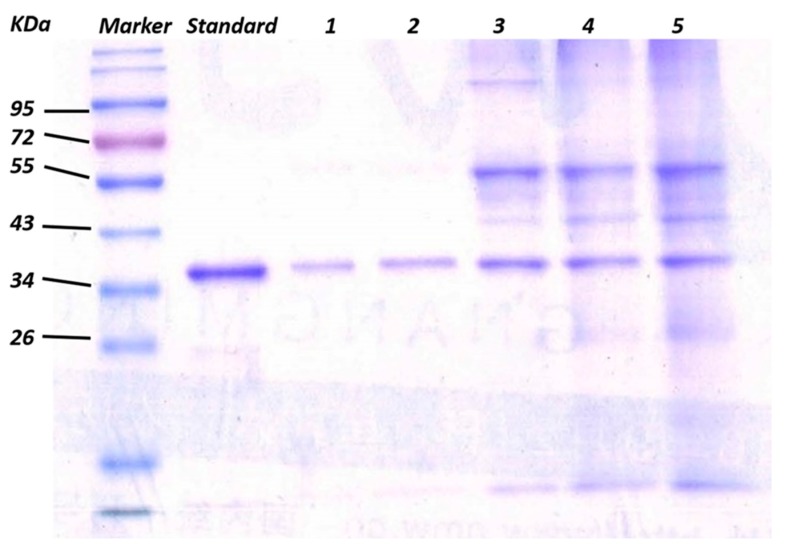
Gel electrophoresis results of captured α-thrombin via GO/Apt nanocomposites from 750 ng of α-thrombin spiked in the buffer solution of BSA with mass ratios of 1:1, 1:10, 1:100, 1:1000 and 1:3000 (lanes 1–5).

For the purpose of evaluating the selectivity of the nanocomposites, different concentrations of BSA were mixed with human α-thrombin in mass ratios from 1:1 to 1:3000. As shown in Figure 5 (lanes 1–5) , the target human α-thrombin could be selectively captured and sufficiently released in SDS–PAGE with an excellent maximum capture efficiency of 95.6%, higher than that of aptamer-modified gold nanorods (84% for 0.98 nM Au) [24] and Au/PEI/GO nanocomposites (86% for 20 μg nanocomposites) [43]. These results have demonstrated that the GO/Apt nanocomposites have high specificity and high efficiency for selectively capturing human α-thrombin, due to the higher amount of immobilized aptamers. Without introducing the component of gold, which mainly results in hydrophobicity that causes nonspecific capture of proteins, the GO/Apt had satisfactory hydrophilicity, which could also explain the performance of this novel nanocomposite.

The effect of the amount of the GO/Apt nanocomposites used in the enrichment of human α-thrombin on the capturing efficiency was also investigated. As shown in Figure S2, five samples of GO/Apt nanocomposites in decreasing concentrations were used to capture a fixed amount (750 ng) of human α-thrombin. The results showed that the capturing efficiency was negatively correlated with the concentration of the nanocomposites, while the human α-thrombin could be efficiently captured even when only 1 μg of GO/Apt was added for enrichment. This could be explained by the distance of the aptamers from the GO surface. When the concentration of GO/Apt increases, there may occur twists of aptamers between the adjacent GO/Apts (because of their flexibility acquired from the thymidine linkers), which might lessen the chance the aptamers have of accessing the target protein. Under lower concentrations, the nanosheets of GO/Apt were not close enough to be twisted around each other, which allowed the nanocomposites to effectively bind the human α-thrombin. The high aptamer coverage of GO/Apt played a predominant role in preserving the high efficiency of enrichment, even when using low concentrations of GO/Apt nanocomposites.

### 3.3. Application of GO/Apt in Human Plasma

**Figure 6:**
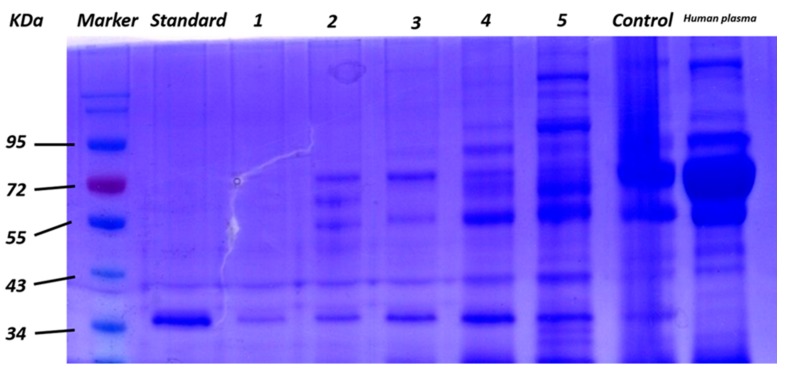
Gel electrophoresis results of captured α-thrombin via GO/Apt nanocomposites from 750 ng of α-thrombin spiked in human plasma concentrations of 0.03 μL/mL, 0.3 μL/mL, 3 μL/mL, 30 μL/mL and 300 μL/mL (lanes 1–5). Standard lane represents 750 ng α-thrombin. Control lane represents 20 μg GO incubated with 750 ng α-thrombin spiked in human plasma with a concentration of 30 μL/mL.

To further evaluate the performance of GO/Apt in a real sample, GO/Apt was employed for the enrichment of human α-thrombin in human plasma samples. In this experiment, different concentrations of human plasma were spiked with 750 ng of human α-thrombin, and separately incubated with GO/Apt nanocomposites under the same conditions. As shown in Figure 6, human α-thrombin could be selectively enriched and detected throughout all concentrations of human plasma, which further demonstrated the excellent selectivity for capturing the target protein. Nonspecific adsorption of proteins was unavoidable under the condition that human plasma was thousands of times more concentrated than that of human α-thrombin. However, the band intensity of the nonspecific proteins was lower than the result of recognition by an Apt-Au NP-contained system [45]. The excessive nonspecific protein adsorption of GO also supported the excellent performance of GO/Apt nanocomposites under extreme conditions of plasma matrix interference. Avoiding the usage of gold nanoparticles in the synthesis of nanocomposites could reduce extra nonspecific adsorption of proteins, which may otherwise be caused by the hydrophobicity of gold nanoparticles. As a result, GO/Apt nanocomposites have been proven to possess great advantages for protein enrichment in real samples.

## 4. Conclusions

A novel, facile one-step synthesis of aptamer-modified graphene oxide nanocomposites was conducted. The nanocomposites were used for the highly specific enrichment of low-abundance human α-thrombin from complex biological samples at the ppb range. The obtained nanocomposites had good hydrophilicity and large coverage of aptamers, which ensured high thrombin capture efficiency even when using very small amounts of material. The pretreatment was rapid, and the enriched protein could be directly analyzed by SDS–PAGE. The excellent capture efficiency (>95%) also outscores other complicated methods that have nonspecific protein adsorption and lower enrichment efficiency. Furthermore, this technique provides a general platform for the enrichment of other proteins, or even cells, by using different aptamers. Quantitative analysis can be also achieved by using an internal standard. Since the band intensity is proportional to the amount of protein, a series of standard thrombin samples in different concentrations could be used in such an experiment, calculating the standard curve as well as other nonspecific proteins. The sensitivity of thrombin could be obtained through the standard curve, and selectivity could be shown according to the significant difference between thrombin and nonspecific proteins. The results obtained here indicate that these aptamer-modified GO nanocomposites are promising materials, and could be applied as simple and efficient sensor probes for protein and cell determination in clinical settings.

## Supplementary Materials

The following are available online at http://www.mdpi.com/1424-8220/17/9/1986/s1, Figure S1: Zeta potentials of GO and GO/Apt. Figure S2: Gel electrophoresis result of captured α-thrombin by 30, 20, 10, 5 μg and 1 μg (lane 1–5) GO/Apt nanocomposites.
