# Supplementary material for: One-Step Facile Synthesis of Aptamer-Modified Graphene Oxide for Highly Specific Enrichment of Human A-Thrombin in Plasma
Source: Sensors (Basel). 2017 Sep 13;17(9):1986. doi: 10.3390/s17091986 (PMC5621013; doi:10.3390/s17091986)
Supplement: Supplementary file 1 [file sensors-17-01986-s001.pdf]

Supporting Information

One-step facile synthesis of aptamer-modified graphene oxide for highly specific enrichment of human α-thrombin in plasma

Yuan Xu, Siyuan Tan, Qionglin Liang*, Mingyu Ding*

Key Laboratory of Bioorganic Phosphorus Chemistry and Chemical Biology, Ministry of Education, Department of Chemistry, Tsinghua University, Beijing 100084, China

***Correspondence:**

Postal address: The Key Laboratory of Bioorganic Phosphorus Chemistry & Chemical Biology, Ministry of Education, Department of Chemistry, Tsinghua University, Beijing 100084, China.

E-mail address: dingmy@mail.tsinghua.edu.cn (Mingyu Ding)

liangql@mail.tsinghua.edu.cn (Qionglin Liang)

Tel: +86-10-62797087 (Ding)；+86-10-62772263 (Liang)

Supporting Figures

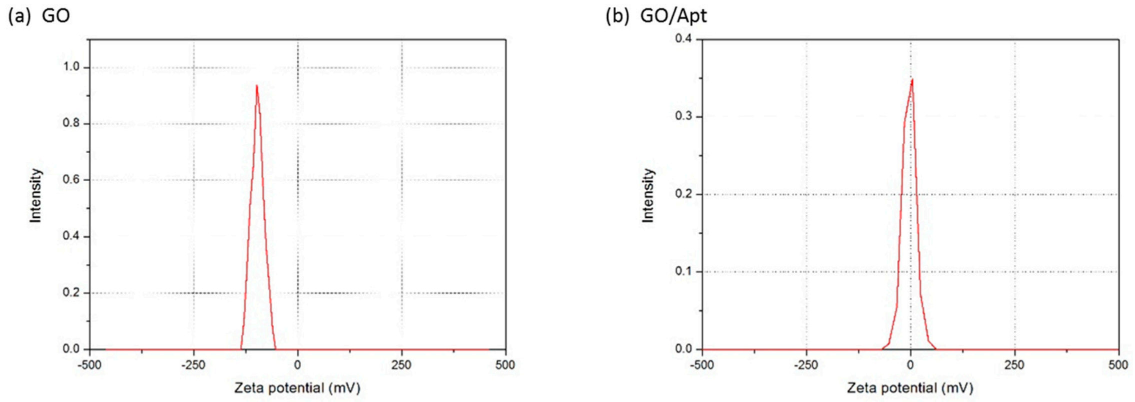

Fig. S1. Zeta potentials of GO and GO/Apt.

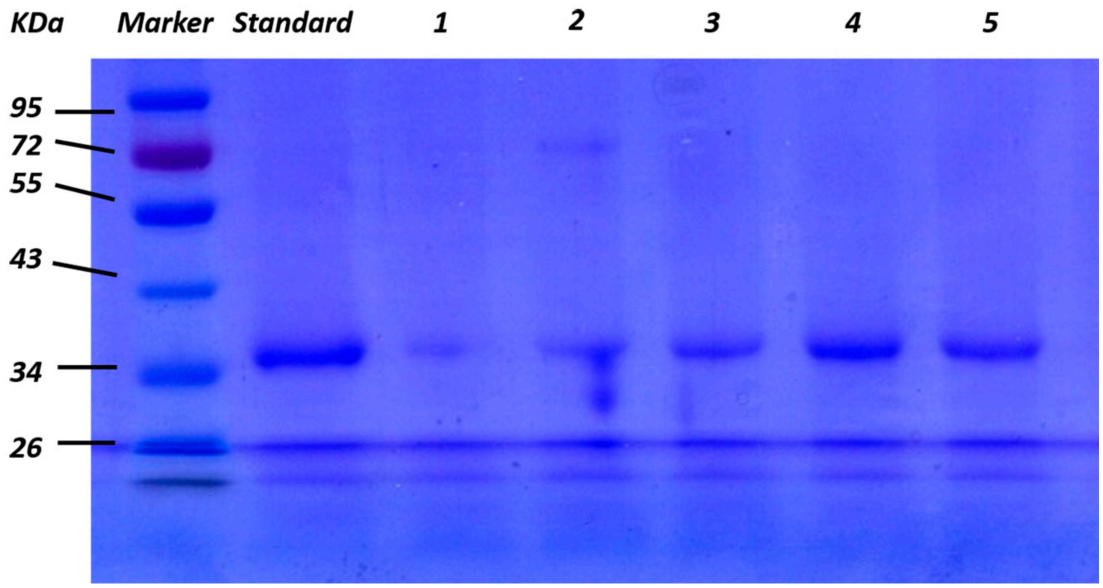

Fig. S2 Gel electrophoresis result of captured α-thrombin by 30, 20, 10, 5 μg and 1 μg (lane 1-5) GO/Apt nanocomposites.
